# Methylmercury Chloride Exposure Affects Oocyte Maturation Through AMPK/mTOR-Mediated Mitochondrial Autophagy

**DOI:** 10.3390/ijms26083603

**Published:** 2025-04-11

**Authors:** Shengkui Hou, Caiyu Wang, Xin Ma, Jing Zhao, Jun Wang, Yi Fang, Hongyu Liu, He Ding, Jing Guo, Wenfa Lu

**Affiliations:** 1 Key Laboratory of the Animal Production, Product Quality and Security, Ministry of Education, Jilin Agricultural University, Changchun 130117, China; shengkuihou@163.com (S.H.); maxin3202@163.com (X.M.); jlndfztd@163.com (J.Z.); junwang2004@126.com (J.W.); fangyi@iga.ac.cn (Y.F.); jlndlhy0133@163.com (H.L.); dinghe1130@126.com (H.D.); 2 Jilin Provincial International Joint Research Center of Animal Breeding and Reproduction Technology, Jilin Agricultural University, Changchun 130117, China; 3 Jilin Provincial Key Laboratory of Beef Cattle Germplasm Resources Conservation and Utilization, Jilin Agricultural University, Changchun 130117, China

**Keywords:** MMC, oocyte, oxidative stress, mitochondrial autophagy

## Abstract

Mercury, a prevalent heavy metal, negatively impacts oocyte maturation. However, the exact mechanism by which methylmercury chloride (MMC) affects this process remains elusive. The present study found that MMC administration triggered meiotic failure in oocytes by disrupting cumulus cell expansion, leading to compromised spindle apparatus and altered chromosomal architecture, which are crucial for oocyte development. This disruption is characterized by abnormal microtubule organization and defective chromosome alignment. Additionally, MMC exposure caused oxidative stress-induced apoptosis due to mitochondrial dysfunction, as indicated by decreased mitochondrial membrane potential, mitochondrial content, mitochondrial DNA copy number, and adenosine triphosphate levels. Proteomic analysis identified 97 differentially expressed proteins, including P62, an autophagy marker. Our results confirmed that MMC induced autophagy, particularly through the hyperactivation of the mitochondrial autophagy to remove damaged and normal mitochondria. The mitochondrial reactive oxygen species (ROS) scavenger Mito-TEMPO alleviated oxidative stress and mitochondrial autophagy levels, suggesting that mitochondrial ROS initiates this autophagic response. Notably, MMC activates mitochondrial autophagy via the monophosphate-activated protein kinase (AMPK)/mammalian target of rapamycin (mTOR) signal pathway due to mitochondrial dysfunction. In vivo studies in mice revealed that MMC exposure decreased reproductive performance, attributed to excessive mitochondrial autophagy leading to reduced oocyte quality. Overall, these findings demonstrate that MMC exposure impairs oocyte maturation via the hyperactivation of mitochondrial autophagy induced by mitochondrial dysfunction.

## 1. Introduction

Mercury, a heavy metal, is widely utilized in various industrial processes, including mining, smelting, the production of pesticides and fertilizers, and medical devices, leading to significant environmental impacts [1,2,3]. Its non-degradability nature allows it to accumulate in humans and mammals through contaminated food and soil [4,5,6]. Mercury is one of the top 10 chemicals that pose serious health risks to humans [7]. Previous studies have shown that circulating mercury can cause abnormal growth or death of nerve cells, leading to chronic nervous system damage [8,9,10]. Methylmercury chloride (MMC) was mainly derived from fish, fungicides, herbicides, marine mollusks, and wood preservatives. MMC exhibits greater toxicity compared to mercuric ions (Hg^2^⁺) due to its enhanced capacity for bioaccumulation [11,12,13]. Furthermore, MMC demonstrates significant reproductive toxicity, manifesting as elevated incidences of menstrual irregularities, reduced fertilization success rates, increased spontaneous abortion rates, and higher stillbirth incidence [14]. Emerging evidence indicates that MMC exposure elicits a dose-dependent progression of follicular atresia while suppressing steroidogenic enzyme activity through epigenetic modifications [15,16]. MMC exposure has been shown to disrupt the development of mouse oocytes [17,18]. Although the toxic effects of MMC on oocyte maturation have been documented, the precise mechanisms by which mercury affects oocytes remain to be fully understood.

Mitochondria, the predominant organelles in oocytes, are essential for adenosine triphosphate (ATP) production and the regulation of intracellular signaling, redox reactions, and apoptosis, all of which contribute to oocyte quality [19]. As a specialized subtype of macroautophagy, mitochondrial autophagy selectively targets and degrades dysfunctional mitochondria through lysosomal pathways, playing an indispensable regulatory role in preserving mitochondrial homeostasis and functional integrity during oocyte maturation [20]. A moderate level of mitochondrial autophagy supports the preservation of mitochondrial quality and physiological function. However, excessive mitochondrial autophagy can significantly increase oxidative byproducts [21,22,23]. In addition, the administration of certain chemicals can mitigate oocyte deterioration by modulating autophagy. Resveratrol has been demonstrated to ameliorate mitochondrial dysfunction associated with post-ovulatory aging through activation of the PTEN-induced putative kinase 1 (PINK1)/Parkin pathway-mediated autophagic processes [24]. Similarly, coenzyme Q10 has been found to delay aging in porcine oocytes by promoting autophagy to eliminate damaged mitochondria. [25]. Conversely, mercury has been identified as a toxic agent that disrupts autophagy. Studies have shown that exposure to methylmercury can induce mitochondrial dysfunction in rat neurons, thereby activating mitochondrial autophagy [26,27].

The present study investigated the effects of methylmercury chloride (MMC) on porcine oocytes by assessing nuclear and cytoplasmic maturation. Our results demonstrated that MMC adversely affects oocyte maturation by disrupting autophagic processes.

## 2. Results

### 2.1. MMC Exposure Impairs the Nuclear Maturation of Porcine Oocyte Maturation

**Figure 1:**
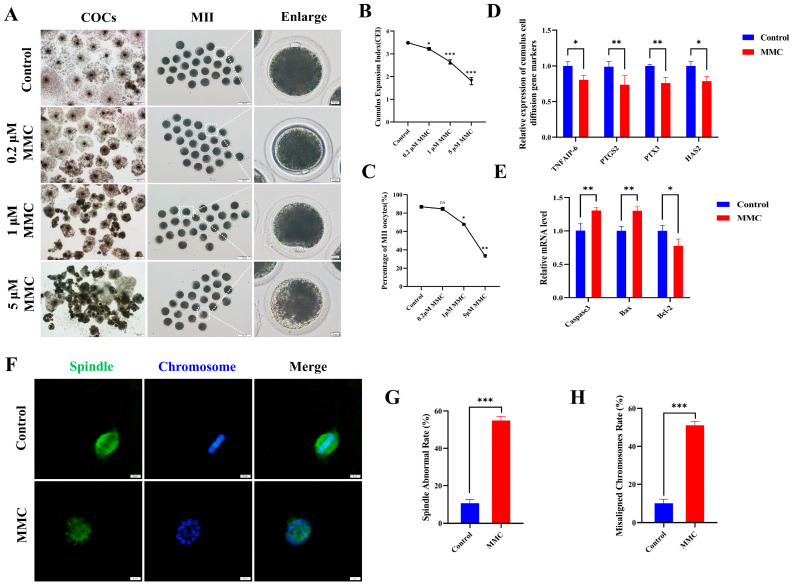
MMC exposure impairs the porcine oocyte maturation. (**A**) Effects of MMC exposure on oocyte cumulus cell expansion and PB extrusion. COCs: scale bar = 200 μm; MII: scale bar = 100 μm; Enlarged: scale bar = 10 μm. (**B**) The cumulus expansion index in control group and MMC group. Control, *n* = 99; 0.2 μM MMC, *n* = 96; 1 μM MMC, *n* = 104; 5 μM MMC, *n* = 101. (**C**) First polar body exclusion rate in control group and MMC group. Control, *n* = 86; 0.2 μM MMC, *n* = 84; 1 μM MMC, *n* = 81; 5 μM MMC, *n* = 91. (**D**) mRNA expression levels of *TNFAIP6*, *PTGS2*, *PTX3*, and *HAS2* were analyzed. (**E**) The apoptosis rate and anti-apoptosis rate of oocytes in control group and MMC group. (**F**) Representative images of spindle morphology and chromosome arrangement after MMC treatment. Scale bar = 5 µm. (**G**) Spindle abnormality rate of oocytes in the control and MMC groups. Control, *n* = 81; MMC, *n* = 83. (**H**) Abnormal chromosome arrangement rate of oocytes in control group and MMC group. Control, *n* = 81; MMC, *n* = 83. ns *p* > 0.05, * *p* < 0.05, ** *p* < 0.01, and *** *p* < 0.001.

To elucidate the effect of MMC on porcine oocyte maturation, we added varying concentrations of MMC (0 μM, 0.2 μM, 1 μM, and 5 μM) to the IVM medium and cultured for 42–44 h. In control cohorts, the majority of oocytes successfully completed first polar body extrusion (PB), whereas experimental groups exposed to MMC administration displayed impaired polar body extrusion capacities (Figure 1A,C). Quantitative analysis revealed a statistically significant concentration-dependent response in PB extrusion rates between intervention groups. Comparative assessments demonstrated marked reductions in extrusion efficiency across MMC-treated oocytes (86.83 ± 1.87% vs. control), manifesting as: 84.68 ± 1.00% (0.2 μΜ, *p* > 0.05), 67.92 ± 2.08% (1 μΜ, *p* < 0.01), and 33.08 ± 0.98% (5 μΜ, *p* < 0.001). As shown in Figure 1A,B, cumulus–oocyte complexes in the control cohort exhibited full cellular expansion, whereas those treated with MMC displayed partial dispersion or complete failure of cellular expansion with statistical significance (*p* < 0.05). Given the significant effects observed at the 1 µM MMC concentration, subsequent experiments were conducted using this concentration. The observed differential downregulation of key cumulus expansion markers (*TNFAIP6 p* < 0.05, *PTGS2 p* < 0.01, *PTX3 p* < 0.01, *HAS2 p* < 0.05) at the transcriptional level provided additional molecular evidence supporting this finding (Figure 1D). Figure 1E demonstrates that MMC treatment markedly upregulated the expression of pro-apoptotic genes *Caspase3* and *Bax* (*p* < 0.01) in cumulus cells, while concurrently downregulating the anti-apoptotic gene *Bcl-2* (*p* < 0.05). These results confirmed the occurrence of apoptosis of cumulus cells. In addition, normal spindle morphology and properly arranged chromosomes are critical criteria for assessing high-quality oocytes. Immunofluorescence analysis of control group oocytes revealed distinct spindle morphology and proper chromosomal alignment, as illustrated in Figure 1F,H. By contrast, the MMC-treated oocytes exhibited a notably higher prevalence of anomalous spindle morphology and dramatically disrupted chromosomal alignment. These results demonstrated MMC treatment impaired porcine oocyte maturation and cumulus cell expansion.

### 2.2. MMC Exposure Causes Oxidative Stress to Induce Early Apoptosis

**Figure 2:**
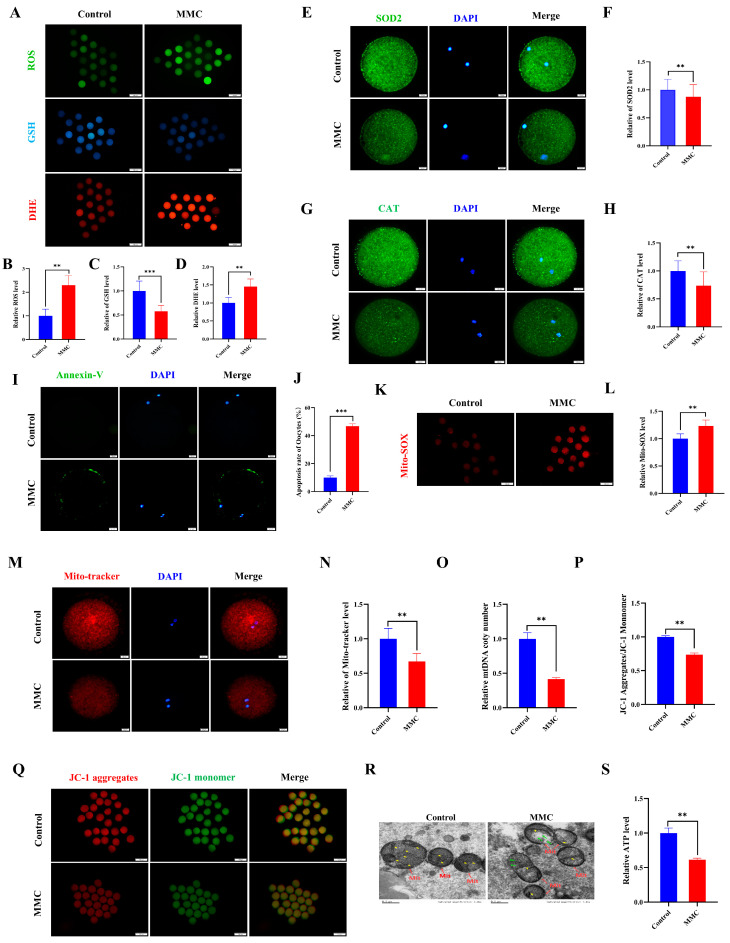
MMC exposure impaired the mitochondrial dysfunction to induce oxidative stress and apoptosis. (**A**) ROS, GSH, and DHE representative images of control group and MMC processing group. Scale bar = 100 μm. (**B**) Relative ROS fluorescence intensity between control group and MMC group. Control, *n* = 82; MMC, *n* = 87. (**C**) Relative GSH fluorescence intensity between control group and MMC group. Control, *n* = 102; MMC, *n* = 98. (**D**) DHE relative fluorescence intensity in control group and MMC group. Control, *n* = 111; MMC, *n* = 107. (**E**) SOD2 representative images of control group and MMC group. Scale bar = 20 μm. (**F**) Relative fluorescence intensity of SOD2 in control group and MMC group. Control, *n* = 88; MMC, *n*= 81. Scale bar = 20 μm. (**G**) CAT representative images of control group and MMC group. Scale bar = 20 μm. (**H**) Relative fluorescence intensity of CAT in control group and MMC group. Control, *n* = 77; MMC, *n* = 79. (**I**) Annexin-V representative images of the control group and the MMC group. Scale bar = 20 μm. (**J**) Apoptosis rate in control group and MMC group. (**K**) Mito-SOX representative images of control group and MMC group. Scale bar = 100 μm. (**L**) Relative fluorescence intensity of Mito-SOX in control group and MMC group. Control, *n* = 75; MMC, *n*= 73. (**M**) Mito-Tracker representative images of control group and MMC group. Scale bar = 20 µm. (**N**) Relative fluorescence intensity of Mito-Tracker in control group and MMC group. Control, *n* = 120; MMC, *n* = 118. (**O**) Relative mtDNA copy number levels between the control group and the MMC group. Control, *n* = 150; MMC, *n* = 150. (**P**) The relative level of ΔΨm (JC-1 aggregate/JC-1 monomer) in control group and MMC group. Control, *n* = 135; MMC, *n* = 129. (**Q**) Representative images of mitochondrial membrane potential in control group and MMC group. Scale bar = 100 µm. (**R**) The control group and MMC group were represented by the mitochondrial morphology of the electron microscope. Red arrowheads represent mitochondria, yellow arrowheads represent mitochondria cristae, and green arrowheads represent the site of mitochondrial damage. Control, *n* = 50; MMC, *n* = 50. Scale bar = 0.5 μm. (**S**) Relative level of ATP in control group and MMC group. Control, *n* = 150; MMC, *n* = 150. ** *p* < 0.01, *** *p* < 0.001.

Current scientific evidence demonstrates a close association between the toxicological effects of mercury exposure and the development of oxidative cellular damage. First, indicators of oxidative stress were detected. Quantitative analysis revealed that oocytes subjected to MMC treatment demonstrated a marked elevation in reactive oxygen species (ROS) and dihydroethidium (DHE) fluorescence intensity compared to control specimens (*p* < 0.01, Figure 2A,B,D). Conversely, glutathione (GSH) concentrations exhibited a significant depletion in the intervention group, reaching statistical significance at *p* < 0.001 (Figure 2A,C). This study examined the expression of superoxide dismutase 2 (SOD2) and catalase (CAT), both of which are key antioxidant enzymes. Notably, both SOD2 and CAT protein levels decreased significantly in the MMC group compared to the control group (*p* < 0.01; Figure 2E–H). These results suggest that MMC induces oocyte oxidative stress by reducing the antioxidant defense capacity of oocytes. As expected, Annexin-V staining was negligible in control oocytes, while a clear fluorescence signal was seen in the MMC group oocytes (Figure 2I). The MMC-treated group showed a higher apoptosis rate (*p* < 0.001; Figure 2J). Notably, oocytes in the MMC group exhibited a pronounced elevation in mitochondrial reactive oxygen species (ROS) fluorescence intensity relative to controls (*p* < 0.01; Figure 2K,L). These observations suggest that MMC-treated oocytes lead to an ROS-induced apoptosis.

Mitochondria are not only energy factories but also the main sites of ROS production. Compared with controls, oocytes in the MMC-exposed group demonstrated a marked reduction in fluorescence intensity (*p* < 0.01; Figure 2M,N), concomitant with significantly diminished mitochondrial DNA copy numbers (*p* < 0.01; Figure 2O). Mitochondrial membrane potential (ΔΨm), a critical parameter for assessing mitochondrial integrity, was substantially compromised following MMC treatment (*p* < 0.01; Figure 2P,Q). The results of transmission electron microscopy (TEM) showed that the mitochondrial morphology of oocytes in the control group was complete; the mitochondrial ridge was clearly visible. In the MMC-treated oocytes, the outer membrane of mitochondria was partially damaged, and some mitochondria cristae were broken and disappeared, forming large vacuoles (Figure 2R). Comparative analysis revealed a marked reduction in porcine oocyte ATP content within the MMC-treated cohort relative to untreated controls (*p* < 0.01), demonstrating that MMC exposure induces mitochondrial dysfunction through oxidative stress potentiation.

### 2.3. MMC Exposure Impaired the Balance of Mitochondrial Autophagy

**Figure 3:**
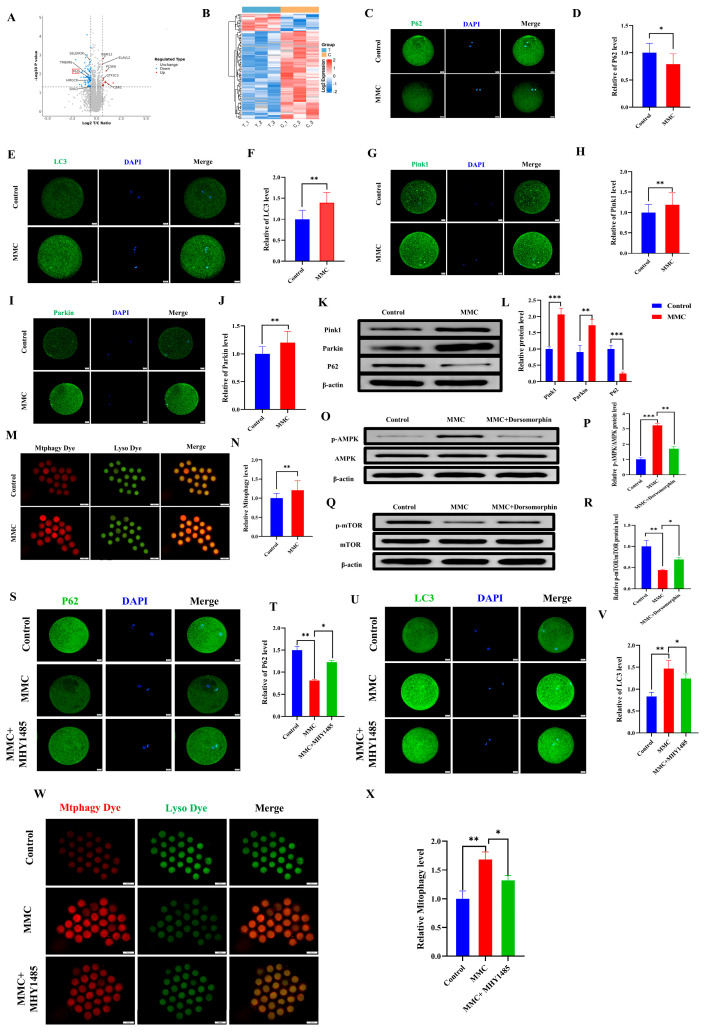
MMC−induced mitochondrial autophagy of porcine oocytes. (**A**) Oocyte differential protein volcano map. When the value was ≥1.50 and *p*-value < 0.05, the differential protein was statistically significant. Red dots represent upregulated proteins, blue dots represent down-regulated proteins, and gray dots show no difference in proteins. The *X*-axis represents fold change, and *Y*-axis means *p*-value. (**B**) Heat map of differential protein cluster expression in porcine oocytes. In the heat map, red represents highly expressed proteins and blue represents low-expressed proteins. (**C**) Representative images of P62 in the control and MMC groups. Scale bar = 20 µm. (**D**) Relative fluorescence intensity of P62 in control group and MMC group. Control, *n* = 77; MMC, *n* = 80. Scale bar = 20 µm. (**E**) Representative images of LC3 in the control and MMC groups. Scale bar = 20 µm. (**F**) LC3 relative fluorescence intensity in control group and MMC group. Control, *n* = 77; MMC, *n* = 79. (**G**) Representative images of Pink1 in the control and MMC treatment groups. Scale bar = 20 µm. (**H**) Relative fluorescence intensity of Pink1 in control group and MMC group. Control, *n* = 85; MMC, *n* = 82. (**I**) Representative images of Parkin in the control and MMC groups. Scale bar = 20 µm. (**J**) Relative fluorescence intensity of Parkin in control group and MMC group. Control, *n* = 86; MMC, *n* = 82. (**K**) The relative protein levels of Pink1, Parkin and P62 were detected by Western blot. (**L**) The protein levels of Pink1, Parkin and P62 were quantitatively analyzed (*n* = 3). (**M**) Representative images of mitochondrial autophagy in control group and MMC-treated group. Scale bar = 100 μm. (**N**) Relative fluorescence intensity of mitochondrial autophagy in control group and MMC treatment group. Control, *n* = 95; MMC, *n* = 89. (**O**) *p*-AMPK, AMPK relative protein level. (**P**) Quantitative analysis of *p*-AMPK and AMPK protein levels (*n* = 3). (**Q**) *p*-mTOR, mTOR relative protein level. (**R**) Quantitative analysis of *p*-mTOR and mTOR protein levels (*n* = 3). (**S**) Representative images of P62. Scale bar = 20 µm. (**T**) The relative fluorescence intensity of P62. Control, *n* = 97; MMC, *n* = 90; MMC + MHY1485, *n* = 95. Scale bar = 20 µm. (**U**) Representative images of LC3. Scale bar = 20 µm. (**V**) LC3 relative fluorescence intensity. Control, *n* = 87; MMC, *n* = 89; MMC + MHY1485, *n* = 89. (**W**) Representative images of mitochondrial autophagy of porcine oocytes in control group, MMC group and MHY1485 treatment group. Scale bar = 100 μm. (**X**) Relative fluorescence intensity of oocyte mitochondrial autophagy in control group, MMC treatment group and MHY1485 treatment group. Control, *n* = 84; MMC, *n* = 83; MMC + MHY1485, *n* = 91, * *p* < 0.05, ** *p* < 0.01, and *** *p* < 0.001.

To investigate the mechanism of MMC on porcine oocytes, an LC-MS/MS proteomic sequence was performed. The analysis identified 97 differentially expressed proteins (DEPs) (fold change > 1.5, *p* < 0.05), including 16 upregulated and 81 downregulated proteins. Autophagy-related protein P62 was included (Figure 3A). The heat map also showed the expression levels of these proteins (Figure 3B). As shown in Figure 3C,D, the P62 staining also confirmed that MMC treatment resulted in the decreased P62 protein expression (*p* < 0.05). Mitochondrial autophagy is the key to maintaining mitochondrial and cell homeostasis [28]. As shown in Figure 3E,F, the MMC group had obvious LC3 points compared with the control group, indicating the formation of autophagosomes (*p* < 0.01). As shown in Figure 3G–J compared with the control group, the expression levels of Pink1 and Parkin in the MMC group were significantly increased (*p* < 0.01). Consistent with this, the Western blot analyses of Pink1, Parkin, and P62 were consistent with those of immunofluorescence staining (*p* < 0.01; Figure 3K,L). Similarly, MMC treatment increased the occurrence of mitochondrial autophagy and the fusion of mitochondria and lysosomes (*p* < 0.01; Figure 3M,N). Energy imbalance activates the AMPK/mTOR pathway, which subsequently induces autophagy. Western blot tests showed that MMC increased the relative protein levels of *p*-AMPK (*p* < 0.001) and decreased the relative protein levels of *p*-mTOR (*p* < 0.01), while Dorsomorphin (AMPK inhibitor) pretreatment reversed these relative protein levels (Figure 3O–R). Immunofluorescence showed that the MMC reduced the relative fluorescence level of P62 (*p* < 0.01), increasing the relative fluorescence level of LC3 (*p* < 0.05), while the mTOR activator could restore the aberrant expression of LC3 and P62 induced by MMC (Figure 3S–V). Importantly, MHY1485 could recover MMC-induced hyperactivation of mitochondrial autophagy (*p* < 0.05, Figure 3W,X). These results showed that mercury treatment could lead to hyperactivation of mitochondrial autophagy via the AMPK/mTOR pathway.

### 2.4. Mito-TEMPO (MT) Mitigated the Negative Effects of MMC Exposure on Porcine Oocyte Quality

**Figure 4:**
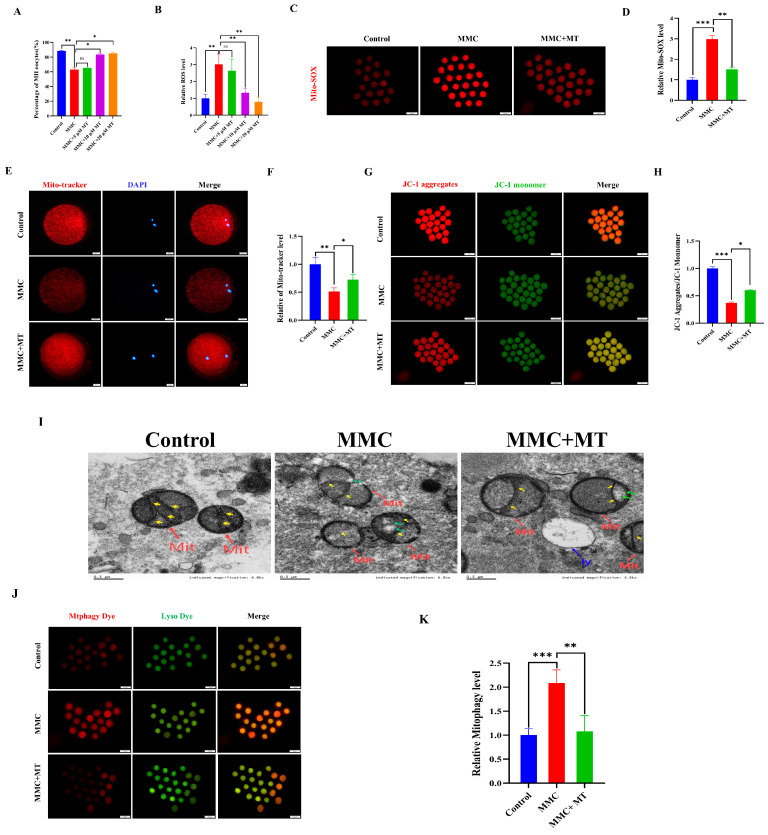
MT alleviated MMC-induced mitochondrial dysfunction of porcine oocytes. (**A**) Effects of different concentrations of MT on porcine oocyte PBI. (**B**) Relative fluorescence intensity of ROS in porcine oocytes with different concentrations of MT. Control, *n* = 102; MMC, *n* = 98; MMC + 5 μM, *n* = 91; MMC + 10 μM, *n* = 95; MMC + 20 μM, *n* = 99. (**C**) Mito-SOX representative images of porcine oocytes in control group, MMC group and MT group. Scale bar = 100 μm. (**D**) Relative fluorescence intensity of Mito-SOX in control group, MMC group and MT treatment group. Control, *n* = 92; MMC, *n* = 89; MMC + MT, *n* = 90. (**E**) Mito-Tracker representative images of porcine oocytes in control group, MMC group, and MT treatment group. Scale bar = 20 μm. (**F**) Relative fluorescence intensity of Mito-Tracker in control group, MMC group and MT treatment group. Control, *n* = 87; MMC, *n* = 91; MMC + MT, *n* = 93. (**G**) Representative images of porcine oocytes ΔΨm in control group, MMC group and MT treatment group. Scale bar = 100 μm. (**H**) The relative fluorescence intensity of ΔΨm in control group, MMC group and MT treatment group. Control, *n* = 90; MMC, *n* = 93; MMC + MT, *n* = 90. (**I**) Transmission electron microscope representative images of mitochondrial morphology in control group, MMC group and MT treatment group. Red arrowheads represent mitochondria, blue arrowheads represent lysosome, yellow arrowheads represent mitochondria cristae and green arrowheads represents the site of mitochondrial damage. Control, *n* = 50; MMC, *n* = 53; MMC + MT, *n* = 50. Scale bar = 0.5 μm. (**J**) Representative images of mitochondrial autophagy of porcine oocytes in control group, MMC group and MT treatment group. Scale bar = 100 μm. (**K**) Relative fluorescence intensity of oocyte mitochondrial autophagy in control group, MMC group and MT treatment group. Control, *n* = 90; MMC, *n* = 93; MMC + MT, *n* = 90. ns *p* > 0.05, * *p* < 0.05, ** *p* < 0.01, and *** *p* < 0.001.

ROS overproduction resulted in the abnormal autophagy. We hypothesize that MMC treatment triggers mitochondrial dysfunction, resulting in excessive accumulation of ROS and subsequent hyperactivation of autophagy. As a mitochondrial superoxide scavenger, MT was used [29,30]. As shown in Figure 4A, 10 µM MT treatment significantly restored the decreased maturation rate of oocytes induced by MMC (Control: 88.44 ± 0.86% vs. 10 µM MT: 83.52 ± 2%; *p* > 0.05). In addition, 10 µM MT treatment also significantly decreased ROS levels compared with the MMC group (*p* < 0.05; Figure 4B). In this study, a concentration of 10 µM MT was selected for subsequent experiments. As shown in Figure 4C,D, the results of Mito-SOX showed that MMC treatment increased the level of Mito-SOX in oocytes (*p* < 0.001), while MT treatment decreased the level of Mito-SOX (*p* < 0.01), indicating that the addition of MT can remove ROS in mitochondria. MMC treatment significantly reduced the level of Mito-tracker (*p* < 0.05; Figure 4E,F), and the Mito-tracker was partially restored after MT treatment (*p* < 0.05). MT treatment also rescued the deceased level of ΔΨm induced by MMC (*p* < 0.01; Figure 4G,H). The results of TEM displayed that MT could rescue MMC-related abnormal morphology of mitochondria (Figure I). Importantly, MT could recover MMC-induced hyperactivation of mitochondrial autophagy (*p* < 0.001, Figure 4J,K). These results suggest that MMC-induced hyperactivation of mitochondrial autophagy was mediated by the excessive accumulation of mitochondrial ROS, and MT recovered these defects.

### 2.5. MMC Exposure Resulted in Impaired Reproductive Performance in Mice

**Figure 5:**
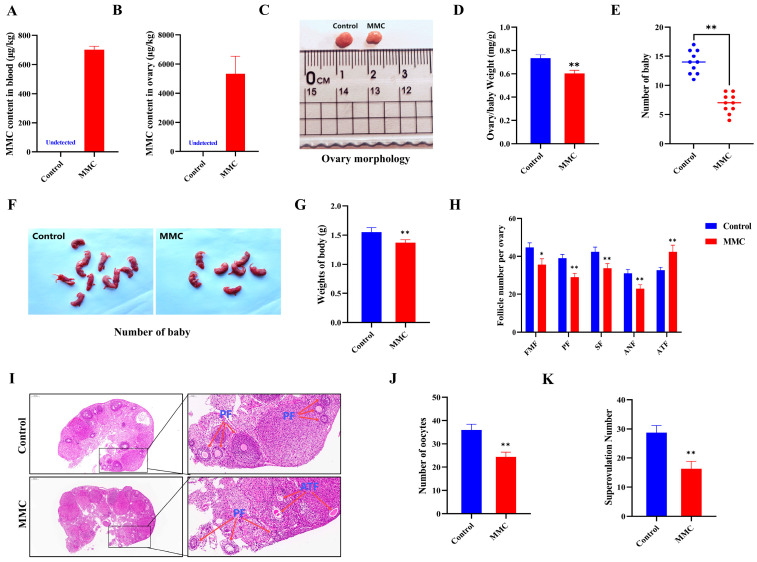
Effects of MMC exposure on reproductive development and offspring quality in mice. (**A**) The amount of MMC in the blood of mice (*n* = 5). (**B**) The content of MMC in mouse ovaries (*n* = 5). (**C**) Representative images of ovarian morphology of mice in control group and MMC treatment group. (**D**) Control group and MMC processing group ovary coefficient (*n* = 10). (**E**) The average litter number of rats in control group and MMC treatment group (*n* = 5). (**F**) Representative images of the number of babies in the control group and the MMC treatment group. (**G**) Average weight of babies in control group and MMC treatment group. (**H**) Follicle count for continuous sections of ovaries (*n* = 5). (**I**) H&E staining representative images of ovary of mice in control group and MMC treatment group. Scale bar = 200 µm; Enlarge: Scale bar = 50 µm. (**J**) Average number of oocytes taken per mouse in control group and MMC treatment group (*n* = 5). (**K**) Ovulated oocytes were counted in control group and MMC group mice. * *p* < 0.05, ** *p* < 0.01.

To assess the impact of MMC on ovarian function in vivo, we quantified reproductive performance in mice following intraperitoneal MMC administration for 7 days. After continuous intraperitoneal injection of MMC for 7 days, MMC content in the blood and ovaries of mice in the MMC group was significantly higher than that in the control group, and MMC was almost not detected in the control group (*p* < 0.001; Figure 5A,B). As shown in Figure 5C,D, compared with the control group, the ovarian coefficient in the MMC group was significantly decreased (control group: 0.73 ± 0.03 mg/g; MMC: 0.6 ± 0.03 mg/g; *p* < 0.01). As shown in Figure 5E–G, the MMC group significantly reduced the number and weight of progeny compared to the control group (*p* < 0.001). In addition, the results showed that MMC treatment caused primitive follicles (FMFs; *p* < 0.05), primary follicles (PFs; *p* < 0.01), secondary follicles (SFs; *p* < 0.01), antral follicles (ANFs; *p* < 0.01), the number of follicles decreased significantly, and the number of atretic follicles increased significantly (ATFs; *p* < 0.05; Figure 5H–I). Importantly, the number of oocytes in mice treated with MMC was significantly lower than that in the control group (*p* < 0.001; Figure 5J). After MMC treatment, the number of mature oocytes in mice decreased significantly. (*p* < 0.001; Figure 5K). These results demonstrated that MMC treatment in vivo impaired the mouse reproductive performance.

### 2.6. MMC Exposure-Impaired Mouse Oocyte Maturation In Vivo

**Figure 6:**
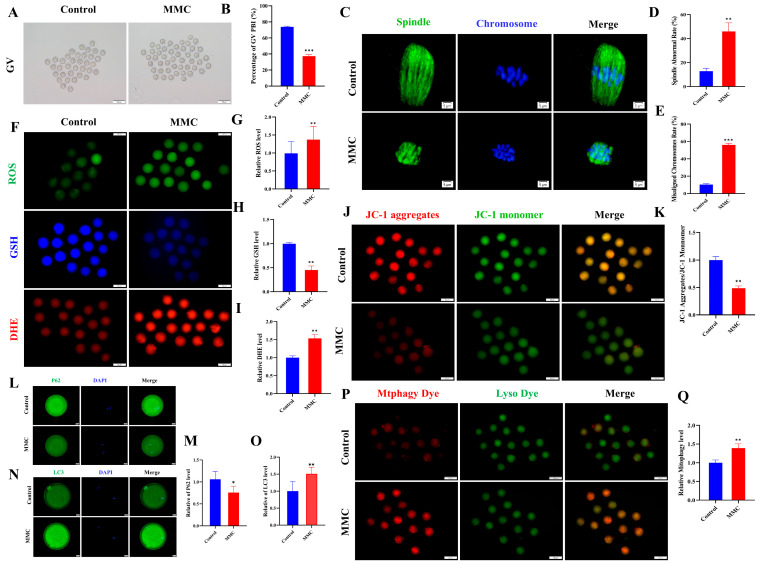
Effect of MMC exposure on oocyte quality in vivo. (**A**) GV oocyte representative images of control group and MMC group. GV: Scale bar = 100 μm. (**B**) GV stage oocyte maturation rate in control group and MMC group. Control, *n* = 93; MMC, *n* = 90. (**C**) Representative images of spindle morphology and chromosome arrangement in the control group and the MMC group. Scale bar = 5 µm. (**D**) Spindle abnormality rate of oocyte in control group and MMC-treated group. Control, *n* = 86; MMC, *n* = 83. (**E**) Abnormal chromosome arrangement rate of oocytes in control group and MMC group. Control, *n* = 90; MMC, *n* = 93. (**F**) Representative images of ROS, GSH, and DHE of oocytes in control group and MMC treatment group. Scale bar = 100 μm. (**G**) Relative fluorescence intensity of ROS in control group and MMC treatment group. Control, *n* = 91; MMC, *n* = 88. (**H**) Relative fluorescence intensity of GSH in control group and MMC group. Control, *n* = 91; MMC, *n* = 88. (**I**) DHE relative fluorescence intensity in control group and MMC treatment group. Control, *n* = 85; MMC, *n* = 87. (**J**) Representative images of ΔΨm in control group and MMC processing group. Scale bar = 100 µm. (**K**) Relative fluorescence intensity of ΔΨm in control group and MMC group. Control, *n* = 104; MMC, *n* = 106. (**L**) Representative images of P62 in control group and MMC group. Scale bar = 20 µm. (**M**) The relative fluorescence intensity of P62 in control group and MMC group. Control, *n* = 92; MMC, *n* = 90. (**N**) Representative images of LC3 in control group and MMC group. Scale bar = 20 µm. (**O**) LC3 relative fluorescence intensity in control group and MMC group. Control, *n* = 101; MMC, *n* = 108. (**P**) Representative images of mitochondrial autophagy in control group and MMC group. Scale bar = 100 µm. (**Q**) Relative fluorescence intensity of mitochondrial autophagy in control group and MMC group. Control, *n* = 93; MMC, *n* = 87. * *p* < 0.05, ** *p* < 0.01, and *** *p* < 0.001.

As shown in Figure 6A,B, GV oocytes from the MMC-treated and saline-treated mice were cultured in vitro to investigate how MMC impaired the mouse oocyte maturation ability. The maturation rate of GV stage oocytes obtained from mice exposed to MMC was significantly lower than that of the control group (control: 73.81 ± 0.87%; MMC: 37.2 ± 2.02%; *p* < 0.001). Spindle morphology and chromosome arrangement were further examined. The results showed that oocytes treated with MMC showed a higher incidence of spindle morphological abnormalities and severe chromosome misalignment (Figure 6C–E). At the same time, immunofluorescence data showed that mercury significantly increased the levels of ROS and DHE in oocytes (*p* < 0.01) and decreased the levels of GSH in oocytes (*p* < 0.01; Figure 6F–I). As shown in Figure 6J,K, MMC treatment significantly reduced the level of ΔΨm (*p* < 0.01). Immunofluorescence staining showed that the expression of the P62 protein decreased after MMC treatment (*p* < 0.05; Figure 6L,M) and LC3 protein levels were significantly increased (*p* < 0.01; Figure 6N,O). Similarly, MMC treatment increased the occurrence of mitochondrial autophagy in mouse oocytes (*p* < 0.01; Figure 6P,Q). These results demonstrated MMC exposure impaired the oocyte maturation ability due to the ROS-induced abnormal mitochondrial autophagy.

## 3. Discussion

Mercury, a prominent chemical linked to health risks, is extensively utilized in various industries [7]. Several studies have shown that environmental pollution increases the risk of reproductive disorders by disrupting oocyte maturation [31,32,33,34]. As a toxic heavy metal, mercury poses reproductive toxicity, yet the exact mechanisms by which mercury affects female germ cells remain elusive. The current study found that exposure to MMC impaired oocyte maturation due to apoptosis induced by excessive mitophagy via the AMPK/mTOR signaling pathway. Moreover, metallothionein (MT) alleviated the negative effects of MMC. In vivo studies also showed that MMC decreased mouse fertility by impairing the oocyte maturation process by activating mitochondrial autophagy.

Furthermore, the effect of MMC on oocytes was evidenced by the decreased polar body extrusion (PBE) rate, a key indicator of oocyte maturation. Cumulus cells, which supply vital growth factors and cytokines for oocyte maturation, showed disrupted communication with oocytes due to MMC, resulting in maturation defects in porcine oocytes [35]. The precise regulation of spindle assembly and chromosome alignment was confirmed through PBE assessment [36]. Additionally, oocytes exposed to MMC exhibited abnormal spindle assembly and misaligned chromosomes. A previous study showed that MMC (1 mg/kg/d) has adverse effects on mouse reproductive performance. However, this study did not detect the effect of MMC on oocytes and the possible mechanism [37]. Our data also revealed that MMC levels were increased in the blood and ovaries of mice after intraperitoneal injection, leading to reduced reproductive performance in female mice, characterized by a rise in atretic follicles and a decrease in the number of offspring. Notably, MMC exposure decreased both oocyte quantity and their developmental potential. Taken together, these findings suggest that MMC adversely affects porcine oocyte maturation in vitro and compromises the quality of mouse oocytes and offspring development in vivo.

Nuclear and cytoplasmic maturation are essential for oocyte maturation. Nuclear maturation can be evaluated by the PBE, spindle morphology, and chromosome alignment. The level of ROS is a key indicator of cytoplasmic maturation, with excessive ROS leading to cellular oxidative stress [38,39]. As anticipated, mercury exposure significantly increased ROS and DHE levels while decreasing GSH, an antioxidant that eliminates ROS [40]. In addition, mercury treatment significantly reduced SOD2 and CAT, major ROS scavengers. These findings suggest that MMC causes excessive ROS accumulation by disrupting cellular mechanisms for ROS elimination. Mitochondria are the main source of ROS production [41,42,43]. The present study found that MMC exposure significantly increased ROS levels in oocytes, impairing mitochondrial function. MT, a ROS scavenger, alleviated the oxidative stress-induced cellular dysfunction [44,45]. Further investigation revealed that treating oocytes with both MT and MMC improved the maturation rate by restoring mitochondrial function. Collectively, these data imply that MMC disrupts oocyte maturation due to the mitochondrial dysfunction induced by ROS accumulation.

Mitochondrial dysfunction can trigger mitochondrial autophagy, a protective mechanism that maintains cellular metabolism by eliminating damaged mitochondria [46]. However, excessive mitochondrial autophagy may create a feedback loop that damages mitochondria and induces apoptosis [47]. The PINK1/Parkin pathway primarily regulates this process [48,49,50]. Previous studies demonstrated that mercury disrupts mitochondrial structure and function by producing ROS and directly binding to key mitochondrial enzymes, leading to mitochondrial depolarization and loss of membrane potential, subsequently activating mitochondrial autophagy [51]. Our study found that MMC triggered mitochondrial autophagy, resulting in apoptosis. Autophagy is a dynamic process categorized into four main stages: energy perception and signal transduction, changes and adjustments in energy levels, the functional phase of signaling molecules, and the self-repair and regulation of energy levels [52]. Therefore, it is inferred that MMC exposure causes damaged mitochondria to activate mitophagy, ultimately leading to apoptosis. 

Proteomic sequencing was performed to further elucidate the mechanism by which MMC affects porcine oocytes. Differential protein screening revealed that MMC exposure significantly reduced the levels of the autophagy marker protein P62, confirming its effect on autophagy. In addition, the result also displayed the increased expression of LC3, which indicated the formation of an autophagosome [53]. The reduction of P62 accompanied by increased LC3 levels indicated that MMC treatment promoted autophagic flux. Meanwhile, MMC exposure increased *p*-AMPK levels in the oocytes, which inhibited *p*-mTOR activity and enhanced mitochondrial autophagy. Treatment with an AMPK inhibitor decreased *p*-AMPK and increased *p*-mTOR protein levels [54]. As the energy sensor, the AMPK pathway was activated due to energy imbalance, stimulating autophagy by inhibiting mTOR, a negative regulator of autophagy [51]. Consequently, the present research demonstrated that MMC induced an energy imbalance due to mitochondrial dysfunction, which activated AMPK and excessively promoted mitochondrial autophagy, ultimately impairing porcine oocyte maturation. Previous research also found that mercury can be detected in the human follicular fluid, which was associated with endometriosis and ovarian response during assisted reproductive technology [55,56,57]. However, the mechanisms underlying mercury-induced ovarian dysfunction remain unclear. We also found that MMC disrupted the mouse reproductive performance, which is related to the hyperactivation of autophagy. This study provides mechanistic insights into how mercury may impair human reproductive health, specifically through dysregulation of autophagy pathways. 

## 4. Materials and Method

### 4.1. Chemicals

All chemicals employed in the experimental procedures were obtained from Sigma-Aldrich (St. Louis, MO, USA) unless stated otherwise. AMPK inhibitor Dorsomorphin (MCE, HY-13418A) was diluted with DMSO, and the concentration of 1 μM was selected for the test according to previous studies [58]. mTOR activator MHY1485 (MCE, HY-B0795) was diluted with DMSO, and a concentration of 200 nM was selected for the test according to previous studies [59].

### 4.2. Collection and Cultivation of Porcine Cumulus Oocyte Complexes (COCs)

Porcine ovaries were aseptically harvested from a local slaughterhouse, maintained in sterile saline (0.9% NaCl) supplemented with antibiotics, and transported to the laboratory in a temperature-controlled container (28–35 °C) within 4 h of collection. Ovarian samples underwent four cycles of lavage using physiological saline containing antibiotic supplementation (75 mg/L penicillin and 50 mg/L streptomycin) to eliminate surface contaminants. Follicles with a diameter of 3–8 mm were selected from the ovarian tissue, and porcine follicle fluid was extracted into a 15 mL centrifuge tube using an 18-gauge needle and a 20 mL syringe. The fluid was left for 10 min to collect the sediment. After natural sedimentation, COCs were resuspended in Tyrode’s lactate-HEPES-polyvinyl alcohol (TL-HEPES-PVA) solution (0.1% polyvinyl alcohol), and the pelleted cellular fraction was aspirated. Cumulus–oocyte complexes (COCs) exhibiting compact cumulus investment and morphologically integral ooplasm were selectively isolated under stereomicroscopic observation. Following sequential triple-wash cycles in TL-HEPES-PVA solution, specimens underwent extended culture (42 h duration) within a tri-gas regulated environment (38.5 °C, 5% CO_2_, saturated humidity). The IVM protocol employed a TCM-199-based formulation enriched with gonadotropic hormones (10 IU/mL PMSG-hCG combination), metabolic modulators (0.1 mg/mL L-cysteine; 0.2 mM sodium pyruvate), mitogenic peptide (10 ng/mL EGF), and biological supplement (10% *v*/*v* autologous porcine follicular fluid).

### 4.3. Mouse Treatment

The 7-week-old SPF mice (ICR) were randomly divided into 2 groups (30 mice per group): the control group (normal saline) and the MMC group. The MMC was dissolved in a saline solution. Both experimental cohorts received equivalent volumes of their respective solutions (saline vehicle for controls versus MMC treatment solution) through standardized administration protocols. The dosage of MMC (1 m/kg⁻^1^/day⁻^1^) was optimized based on prior validated pharmacokinetic protocols [60]. In this study, all mice were raised in a special room with 12 h of light per day, a temperature of 22 ± 1 °C, and a relative humidity of 55 ± 5%. They were provided with sterilized pelleted feed and water via automated delivery systems to ensure constant access to nutrition. After a week of intraperitoneal injections, blood and ovarian tissues were collected. Blood samples were collected after anesthesia, and serum was obtained through centrifugation. Each serum sample volume was 350 μL and stored at 4 °C.

### 4.4. Hematoxylin and Eosin (H&E) Staining

Histological sections underwent a sequential deparaffinization process with three xylene immersions, following standard histochemical protocols. Sections were rehydrated for 2 min through a series of ethanol concentrations (100, 90, 80, and 70%), followed by a 5-min rinse in distilled water. For nuclear staining, the sections were treated with Harris hematoxylin (Sigma-Aldrich, H3136, St. Louis, MO, USA) for 5 min and rinsed under running water for another 5 min. Tissue specimens were sequentially processed as follows: (1) acidic differentiation in 1% hydrochloric acid (HCl)/70% (*v*/*v*) ethanol (30-s immersion) to enhance the nuclear-cytoplasmic contrast; (2) hydration via continuous aqueous irrigation (1 min; 25 °C) to eliminate residual dye aggregates, following standard histochemical protocols. To optimize azurophilic staining enhancement, tissue sections were subjected to 0.1% ammonium hydroxide solution or Scott’s sodium bicarbonate-based tap water substitute (pH 8.0) for precisely 60 sec, followed by thorough rinsing in deionized H_2_O. Subsequent cytoplasmic counterstaining was achieved using a 0.5% eosin Y solution (Sigma-Aldrich, 105174, St. Louis, MO, USA) with a controlled immersion duration of 120 ± 5 s, after which residual dye was eliminated through three cycles of distilled water washing. Finally, histological preparations underwent sequential dehydration in an ascending ethanol gradient (70% → 80% → 90% → absolute ethanol), with each step maintained for 120 sec under controlled temperature conditions (23 ± 1 °C). Transparency was achieved via a dual-phase solvent exchange in xylene (2 × 5-min cycles at ambient temperature). The stained slides were examined under an optical microscope, and a digital camera was used to shoot.

### 4.5. Mice Oocyte Collecting and Culture

Mice were injected intraperitoneally with 10 IU PMSG, euthanized 48 h later, and GV stage oocytes were collected. GV oocytes were cultured for 16 h in the M2 medium. Mice were intraperitoneally injected with 10 IU PMSG and then injected with 10 IU hCG (NSHF, Ningbo, China) 48 h later to collect MII oocytes. The superovulated mice were euthanized after 13 h of hCG injection, and the oviductal ampullae were punctured with a syringe to release COCs. Cumulus-denuded oocytes were obtained by transferring the COCs into a phosphate-buffered saline (PBS) medium containing 0.1% hyaluronidase and gently pipetting cumulus cells.

### 4.6. Treatment of MMC 

MMC (Aladdin, 115-09-3) was diluted to 0.2, 1, or 5 μM in TCM-199 for the subsequent culture test.

### 4.7. Evaluation of Cumulus Expansion

The COCs were cultured in a cell incubator for 42 h, and the cumulus cell expansion index (CEI) was calculated according to referenced materials [61]. 

### 4.8. Analysis of Reactive Oxygen Species (ROS), Mitochondrial Superoxide Indicators (MitoSOX), Mitochondrial Membrane Potential (∆ψm), Mitochondria Morphology Glutathione (GSH), Dihydroergotamine (DHE), Autophagy Level, and Apoptosis

Following a 42–44 h culture period under standard incubation conditions (37°C, 5% CO_2_), cumulus–oocyte complexes underwent enzymatic denudation using 0.2% hyaluronidase. Metaphase II (MII) oocytes were subsequently subjected to fluorophore labeling, with quantitative fluorescence detection performed under calibrated microscopic parameters.

The oocytes underwent fluorescent probe staining under standardized conditions (37 °C, 5% CO_2_, 30 min), using the following reagents: JC-1 (2 µM; Beyotime, Shanghai, China, Cat#C2006) for ΔΨm quantification; dichlorodihydrofluorescein diacetate (DCFH-DA, 10 µM; Beyotime, Cat#S0033S) for ROS detection; CellTracker™ Blue (10 µM; Invitrogen, Carlsbad, CA, USA, C12881) for GSH assessment; dihydroethidium (DHE; 10 µM; Beyotime, Cat#S0061) for superoxide anion evaluation; Annexin-V/propidium iodide (PI) dual staining (Beyotime, Cat#C1062L) for apoptotic index determination; MitoSOX™ Red (10 µM; MedChemExpress, Monmouth Junction, NJ, USA, HY-D1056) for MitoSOX quantification; MitoTracker™ Red CMXRos (10 µM; Beyotime, Cat#C1049B) for mitochondrial network visualization; and Mtphagy Dye (1 µM; Dojindo, Kumamoto, Japan MD01) for mitophagy flux analysis. The fluorescent probes were incubated with samples under temperature-controlled conditions (38.5 ± 0.2°C, 30 min), followed by thorough PBS rinsing (3 times for 5 min each) to eliminate unbound dyes. Fluorescence quantification was executed on an Olympus IX83 inverted fluorescence microscope integrated with CellSens Dimension 3.2 software (v1.18) under standardized acquisition parameters: JC-1 monomer/aggregate at 488/530 nm vs. 529/590 nm excitation/emission; ROS at 488/530 nm; GSH at 371/464 nm; and DHE and mitochondria at 529/590 nm.

### 4.9. Measurement of ATP Content

ATP levels in oocytes (*n* = 50 per group) were quantified using a chemiluminescent ATP assay kit (Abcam, Cambridge, UK, ab83355)according to the manufacturer’s protocol, with luminescence measurements recorded via a luminometer plate reader (wavelength range of 400–600 nm).

### 4.10. Transmission Electron Microscopy

Oocytes were initially immobilized in a 2.5% glutaraldehyde solution at 4 °C for 2 days. They were then treated with a 1% agar solution for 40 min, followed by a gradual dehydration process with ethanol. Next, the oocytes were soaked in propylene oxide for solvent exchange, embedded in Epon 812, and sectioned. Ultrathin sections (60–80 nm in thickness) were prepared using ultramicrotome-equipped diamond knives, affixed to 200-mesh copper grids, and subsequently subjected to dual-staining protocols involving aqueous uranyl acetate and Reynolds’ lead citrate. A 200 kV FEI Tecnai 8482 electron microscope was applied for sample examination and imaging.

### 4.11. Immunofluorescence Staining

After removing the cumulus cells from the oocytes, the oocytes were cleaned thrice with PBS-polyvinylpyrrolidone (PVP), fixed with 4% paraformaldehyde for 30 min, and permeated with 0.2% Triton X-100 solution for another 30 min. Following three washes in phosphate-buffered saline (PBS), oocytes were blocked with 3% (*w*/*v*) bovine serum albumin (BSA) in PBS for 1 h at room temperature, followed by incubation with primary antibodies diluted in blocking buffer at 4 °C for 12–16 h. The next day, cells were subjected to three 5 min washes in PBS containing 0.1% polyvinylpyrrolidone (PBS-PVP) and subsequently incubated with species-appropriate fluorescent secondary antibodies (1:400) for 1 h at ambient temperature protected from light. Nuclear counterstaining was performed using 4′, 6-diamidino-2-phenylindole (DAPI; 1 μg/mL) for 10 min, followed by three additional PBS-PVP washes. Specimens were mounted under #1.5 coverslips using ProLong Gold Antifade Mountant and imaged using an Olympus BX51 epifluorescence microscope (Tokyo, Japan) equipped with a cellSens Standard imaging system.

The antibody specifications were as follows:

- α-tubulin-FITC (1:50; Beyotime, Cat# AF0001);
- LC3B (1:100; Abcam, Cat# SAB5600232);
- Tom20 (1:100; Proteintech, Rosemont, IL, USA, Cat# 66504-1);
- CAT (1:100; Proteintech, Cat# 21260-1-AP);
- SOD2 (1:100; Proteintech, Cat# 24127-1-AP);
- PINK1 (1:100; Proteintech, Cat# 66504-1);
- Parkin (1:100; Proteintech, Cat# 66504-1);
- SQSTM1/P62 (1:100; Abcam, Cat# ab207305);
- Alexa Fluor 488-conjugated secondary antibodies (1:400; Invitrogen, Goat anti-rabbit Cat# A-11008; Goat anti-mouse Cat# A-11001).

### 4.12. Mitochondrial DNA (mtDNA) Copy Number Detection

Mitochondrial DNA (mtDNA) copy number quantification was performed according to established protocols [62]. Fifty randomly selected oocytes were enzymatically isolated and resuspended in 2 μL phosphate-buffered saline (PBS) for nucleic acid isolation. The cellular suspension underwent centrifugation (4 °C, 12,000× *g*, 30 s) followed by the addition of 8 μL 5 mM Tris-HCl buffer (pH 8.0). Samples were subjected to thermal denaturation at 95 °C for 10 min, then supplemented with proteinase K (20 mg/mL) for enzymatic digestion at 55 °C for 30 min. Enzyme inactivation was achieved through subsequent incubation at 95 °C for 10 min. The resultant lysate was analyzed using SYBR Green-based quantitative PCR with a Rotor-GeneTM 6000 real-time PCR system (Corbett Research), employing specific primer sets for mtDNA quantification.

### 4.13. Western Blotting

Porcine oocytes (*n* = 50 per experimental group) were subjected to enzymatic zona pellucida removal through pronase digestion (0.5% *w/v*). Following three successive washes in SOF-HEPES buffer (37 °C, 5% CO_2_), cellular lysates were prepared using RIPA buffer supplemented with protease–phosphatase inhibitor cocktail (44:1:5 *v*/*v*) through mechanical homogenization. Protein denaturation was achieved via 10 min incubation in a 95 °C metal thermal cycler. Electrophoretic separation was performed on 12% SDS-polyacrylamide gels under reducing conditions, followed by semi-dry electrotransfer (25 V, 30 min) to PVDF membranes pre-blocked with 5% BSA. Membranes were probed with primary antibodies at 4 °C for 16 h using the following specifications:

Autophagy markers: LC3B (Abcam SAB5600232; 1:1000), PINK1 (Proteintech 66504-1; 1:1000), Parkin (Proteintech 66504-1; 1:1000), SQSTM1/P62 (Abcam ab207305; 1:1000), Metabolic regulators: AMPK (CST D62G4; 1:1000), phospho-AMPKThr172 (CST 2535; 1:1000), mTOR (Proteintech 66888-1; 1:1000), phospho-mTORSer2448 (Proteintech 67778-1; 1:1000), Loading control: β-actin (CST 4970; 1:1000), Post three TBST washes (10 min/wash), membranes were incubated with species-matched HRP-conjugated secondary antibodies (1:5000) for 1 h at room temperature. Chemiluminescent signals were detected using ECL Prime substrate (Cytiva, Marlborough, MA, USA, RPN2232) and quantified using Amersham Imager 600 with ImageQuant TL software (v8.1). All immunoblotting experiments included three biological replicates with technical triplicates.

### 4.14. RNA Isolation, Complementary DNA (cDNA) Synthesis, and Quantitative Reverse Transcription-PCR (qRT-PCR) 

The expression levels of *TNFAIP6*, *PTGS2*, *PTX3*, *HAS2*, *Caspase3*, *Bax*, *Bcl-2*, and *ATP6* were quantified using quantitative reverse transcription-polymerase chain reaction (qRT-PCR) analysis. RNA was extracted using the Arcturus PicoPure RNA separation kit (Thermo, Waltham, MA, USA, KIT0204). cDNA synthesis was performed using One-Step gDNA Removal (TransGen, AE341-02)and cDNA Synthesis SuperMix (TransGen, Beijing, China, AE301-02). The quantitative reverse transcription PCR (qRT-PCR) was performed in a 20 μL reaction system containing 1 μL of template cDNA, 10 μL of SYBR Green Premix Ex Taq™, 0.8 μL of gene-specific forward/reverse primers (10 μM), 0.4 μL of ROX Reference Dye II, and 7.8 μL of nuclease-free water. All primer pairs were experimentally validated for amplification efficiency and specificity prior to formal experiments, with their corresponding nucleotide sequences and GenBank accession numbers systematically cataloged in Table 1.

### 4.15. Proteomics

#### 4.15.1. Protein Extraction

Oocytes were cultured for 42–44 h before cumulus cells were removed. Fifty oocytes from each group were then treated with high-intensity ultrasound in a lysate. Following centrifugation under refrigerated conditions (4 °C) at 12,000× *g* for 10 min, cellular debris was separated from the protein-rich supernatant. Experiments were performed in triplicate for both the control and MMC groups.

#### 4.15.2. Trypsin Digestion

An equal amount of protein from the control and MMC groups was digested. The resultant liquid was adjusted to the same volume as the solution, and then the dithisusacchariol was added to achieve a final concentration of 5 mM. A reduction reaction was performed at 56 °C for 30 min. Then, a specific amount of iodoacetamide was added, increasing the concentration to 11 mM, and the mixture was incubated at room temperature for 15 min in the dark. Subsequently, teab was added to dilute the urea, keeping the concentration below 2 M. Protease and protein were mixed in a ratio of 1:50 and allowed to react overnight. Meanwhile, a 1:100 ratio of protease-to-protein continued the enzymatic reaction.

#### 4.15.3. Database Search

Data-independent acquisition (DIA) datasets were analyzed using the DIA-NN computational platform (version 1.8). Spectral matching was performed against the Sus_scrofa_9823_PR_20231009 protein sequence database (comprising 46,179 entries), along with its corresponding reverse decoy dataset. Peptide identification employed trypsin/P as the proteolytic enzyme, allowing for a miscleavage. Protein modifications included the removal of invariant methionine at the N-termini and fixed carbamidomethylation at cysteine residues. A stringent false discovery rate threshold (≤1%) was rigorously enforced across all detected peptide species to ensure statistical confidence in identifications.

### 4.16. Statistical Analysis 

Experimental datasets were derived from a minimum of three biologically independent replicates and presented as mean values ± standard error of the mean (SEM). Statistical analyses were performed using SPSS (version 20.0; SPSS Inc., Chicago, IL, USA) and GraphPad Prism software packages (version 6.01; La Jolla, CA, USA), with ImageJ (version 18.0; GraphPad, NIST, USA) utilized for comprehensive data analysis and graphical representation. Following verification of data distribution normality and homogeneity of variance through appropriate diagnostic testing, non-parametric datasets were subjected to Kruskal–Wallis analysis while parametric data underwent one-way ANOVA processing. Post hoc comparisons were conducted using Duncan’s multiple range test to establish statistically significant differences between experimental groups.

## 5. Conclusions

In summary, this research demonstrated that exposure to MMC induced mitochondrial dysfunction, leading to oxidative stress-induced apoptosis. The mitochondrial dysfunction resulted in MMC-induced hyperactivation of mitochondrial autophagy through the AMPK/mTOR pathway, aimed at eliminating both damaged and normal mitochondria, ultimately leading to the arrest of porcine oocytes. These findings deepen our understanding of the negative effects of mercury on the female reproductive system, particularly regarding oocyte maturation.

## Figures and Tables

**Table 1:** Primer sequence information.

Genes	Primer Sequences (5′–3′)	
	Forward	Reverse
*18S*	CGCGGAAGGATTTAAAGTG	AAACGGCTACCACATCCAAG
*TNFAIP6*	GCGAAAGCGGTGTGTGAATACG	CCAACTCTGCCCTTAGCCATCC
*PTGS2*	GCGAGGACCAGATTTCACGA	CAGACCAGGCACCAGACCAA
*PTX3*	CCCGGCAACACCAATGAAAC	CGTTGACCCACAAGGCTACA
*HAS2*	CTCGGTCCAAGTGCCTTACTGAG	TGCCTCATAGGTCATCCACAAGTG
*Caspase3*	GTGGGATTGAGACGGACAGTGG	TTCGCCAGGAATAGTAACCAGGTG
*BAX*	CACCAAGAAGCTGAGCGAGTGT	TCGGAAAAAGACCTCTCGGGGA
*Bcl-2*	TGGATGACCGAGTACCTGAA	CAGCCAGGAGAAATCAAACA
*ATP6*	TATTTGCCTCTTTCATTGCCC	GGATCGAGATTGTGCGGTTAT

## Data Availability

The data that support the findings of this study are available from the corresponding author upon reasonable request.
